# Designing Socially Assistive Robots for People With Alzheimer’s and Related Dementia

**DOI:** 10.1093/geroni/igab046.1550

**Published:** 2021-12-17

**Authors:** Kimberly Mitchell, Xiaopeng Zhao, Jinxiao Yu, Luke MacDougall

**Affiliations:** 1 University of Tennessee, Knoxville, Tennessee, United States; 2 University of Tennessee, University of Tennessee, Tennessee, United States; 3 University of Tennessee, Knoxville, Knoxville, Tennessee, United States; 4 University of Tennessee - Knoxville, Knoxville, TN, Tennessee, United States

## Abstract

Alzheimer’s disease (AD) is the most common form of dementia and is associated with memory loss and cognitive impairments that affect daily life. Approximately 5.8 million older adults in the U.S. are living with AD. People with AD often require high levels of care and assistance to maintain daily activities. The majority of care provided to a person living with AD or other forms of dementia is from a family caregiver, representing 18.6 billion hours of unpaid care valued at $244 billion. The long duration, time-intensive nature of caregiving imposes high burdens on caregivers. We designed a socially assistive robot to engage in conversation with people with AD, by engaging in conversation, helping them conduct simple daily tasks, and relieving caregivers of some of their responsibilities. Using design-thinking methodology, a prototype social robot has been created using 3-d printing technology and a single board computer based on raspberry Pi. Interaction between human and robot is implemented using the Mycroft open source voice assistant. The authors demonstrated that the robot is able to have natural conversations with human users. The overall cost of the robot is estimated to be less than $300, rendering it possible for wide distribution among the public. Future research includes further implementation of various cognitive assessment and cognitive training programs using the social robot to improve the quality of life for people living with AD.

